# A multi‐class COVID‐19 segmentation network with pyramid attention and edge loss in CT images

**DOI:** 10.1049/ipr2.12249

**Published:** 2021-05-04

**Authors:** Fuli Yu, Yu Zhu, Xiangxiang Qin, Ying Xin, Dawei Yang, Tao Xu

**Affiliations:** ^1^ School of Information Science and Engineering East China University of Science and Technology Shanghai 200237 People's Republic of China; ^2^ Department of Endocrine and Metabolic Diseases The Affiliated Hospital of Qingdao University Qingdao 266003 People's Republic of China; ^3^ Department of Pulmonary Medicine Zhongshan Hospital Fudan University Shanghai 200032 People's Republic of China; ^4^ Department of Pulmonary and Critical Care Medicine The Affiliated Hospital of Qingdao University Qingdao Shandong 266000 People's Republic of China

**Keywords:** X‐rays and particle beams (medical uses), Patient diagnostic methods and instrumentation, Optical, image and video signal processing, Image recognition, X‐ray techniques: radiography and computed tomography (biomedical imaging/measurement), Computer vision and image processing techniques, Biology and medical computing

## Abstract

At the end of 2019, a novel coronavirus COVID‐19 broke out. Due to its high contagiousness, more than 74 million people have been infected worldwide. Automatic segmentation of the COVID‐19 lesion area in CT images is an effective auxiliary medical technology which can quantitatively diagnose and judge the severity of the disease. In this paper, a multi‐class COVID‐19 CT image segmentation network is proposed, which includes a pyramid attention module to extract multi‐scale contextual attention information, and a residual convolution module to improve the discriminative ability of the network. A wavelet edge loss function is also proposed to extract edge features of the lesion area to improve the segmentation accuracy. For the experiment, a dataset of 4369 CT slices is constructed, including three symptoms: ground glass opacities, interstitial infiltrates, and lung consolidation. The dice similarity coefficients of three symptoms of the model achieve 0.7704, 0.7900, 0.8241 respectively. The performance of the proposed network on public dataset COVID‐SemiSeg is also evaluated. The results demonstrate that this model outperforms other state‐of‐the‐art methods and can be a powerful tool to assist in the diagnosis of positive infection cases, and promote the development of intelligent technology in the medical field.

## 1 INTRODUCTION

At the end of 2019, a novel coronavirus named COVID‐19 (Corona Virus Disease 2019) broke out and made people panic around the world [1, 2]. The clinical manifestations of the disease are mainly fever, fatigue, and dry cough, severe patients may have difficulty breathing, multiple organ failure or even death. And it can be spread through respiratory, droplets and contact, so it is highly infectious [3]. As of 12:26 PM on 18 December, 2020, there have been a total of 74,921,288 infections and 1,661,789 deaths worldwide [4]. At present, COVID‐19 has been well controlled in China, but there are occasional emergencies in some areas. In this case, we need to efficiently detect the new coronavirus and isolate patients in time to limit the spread of the virus. For the diagnosis of COVID‐19, the current golden standard method is Reverse Transcription Polymerase Chain Reaction (RT‐PCR). However, the RT‐PCR technology is time‐consuming and the medical equipment is also insufficient [5]. Therefore, a quick and effective auxiliary diagnosis technology is helpful to the current situation.

**FIGURE 1:**
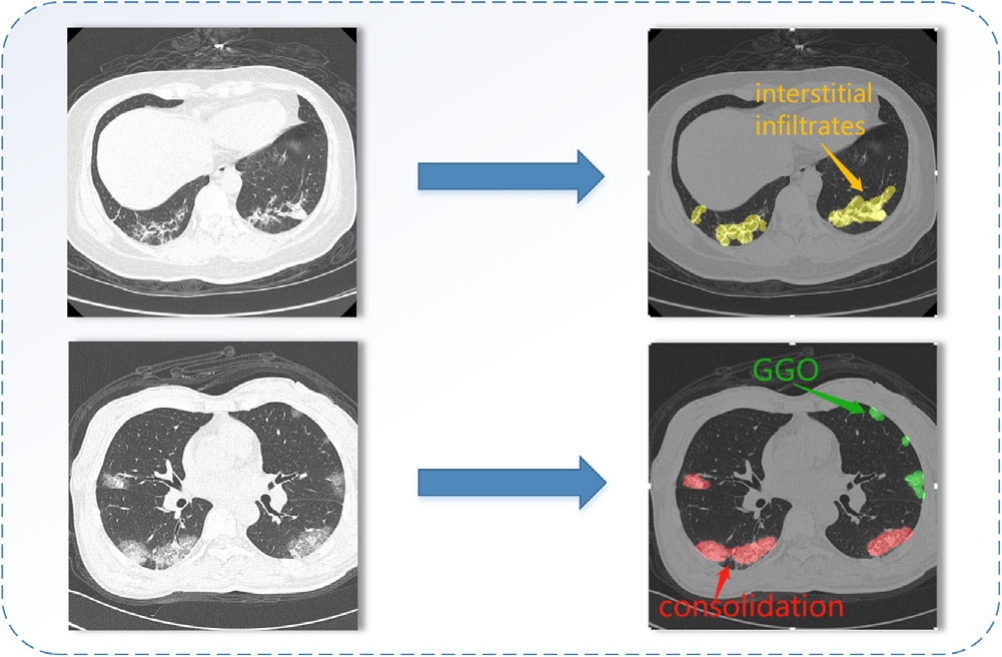
Examples of COVID‐19 infected regions in CT slices, where green, yellow, red denote the GGO, interstitial infiltrates and consolidation respectively

With the development of science and technology, medical imaging technology has been widely used in clinical practice. It can accurately locate, qualitatively and quantitatively diagnose diseases [6]. Research has found that COVID‐19 can show symptoms in radiological imaging such as Computed Tomography (CT) and X‐ray, and the combination of laboratory results and radiological features is helpful in the diagnosis of COVID‐19 [7]. CT is an effective auxiliary diagnostic technology when the test kits are insufficient, it is less time‐consuming, and the work of [8] states that CT scan tests can be more sensitive than RT‐PCR. Radiological CT images contain many useful information and present symptoms. In [9], 114 COVID‐19 cases were collected, and the study found that most patients had multi‐lobe lesions, 30 patients had ground glass opacities, 30 patients had the consolidation change, and 50 cases had both symptoms. Li et al. in [10] analysed 131 confirmed cases in Southwest China, they found that 109 (83%) cases showed more than two lobes involved, 20 (15%) cases had patchy ground glass opacities. Yoon et al. [11] showed that COVID‐19 pneumonia in Korea mainly presented pure to mixed ground glass opacities in a patchy or nodular shape. Ref. [12] found that COVID‐19 lesions could quickly change into the diffuse ground glass opacity mainly or consolidation pattern in 1–3 weeks. In this paper, we detect three types of lesions: ground glass opacities, interstitial infiltrates and consolidation, and they are labelled with green, yellow, red, respectively, as shown in Figure 1. In the early stage, chest CT shows single or multiple scattered patchy ground glass opacities, which are mainly distributed in the middle and lower lung and bronchial vascular bundles. In the advanced stage, the symptom of diffuse consolidation of varying density will present in CT imaging. And in the recovery stage, chest CT images will show interstitial infiltrate symptom. Therefore, we can judge the severity of the patient's condition.

Traditional medical imaging diagnosis relies on the level and experience of professional doctors. There are problems such as strong subjectivity, low repeatability and insufficient quantitative analysis, so intelligent technology is needed to help doctors improve the accuracy and efficiency of diagnosis. With the rapid development of artificial intelligence, deep learning methods have been applied in many medical fields such as cancer detection [13, 14], lung segmentation [15, 16]. Recently, deep neural segmentation networks have been proposed for the detection of COVID‐19 [39, 40, 41, 42, 43, 44, 45, 46]. However, it is still challenging to detect the COVID‐19 lesion area through CT images [17]. Firstly, the texture, shape, and location of the infected area are prone to change, which brings difficulties to detection. Secondly, the boundary of the infected area is fuzzy, irregular, and low contrast, which brings challenges to accurate segmentation.

To address above problems, this paper proposes a novel COVID‐19 segmentation network that can locate the lesion area in CT slices. We employ the resnet101 as the backbone [18], and propose a pyramid attention module which applies a multi‐scale channel attention mechanism with the fusion of maximum and average pooling to enhance the feature expression. And we construct a residual convolution module based on the residual block of resnet101 to extract deep semantic information. Due to the complex contour of the COVID‐19 lesion area, we propose a new loss function called wavelet loss to achieve more accurate segmentation. Wavelet decomposition can extract high‐frequency information in multiple directions of the image [19], so we use wavelet transformation to obtain multi‐angle edge information of the lesion area. The wavelet‐based edge loss function can focus on irregular boundaries, thereby improving the performance of the model. The main contributions of this paper are as follows:

1. We propose a novel multi‐class COVID‐19 segmentation network which can more accurately locate the lesion area in CT images. It is a classic encoder‐decoder structure, and we design a pyramid attention mechanism and a new loss function based on wavelet decomposition to improve the performance of the model.
2. The proposed pyramid attention module combines pyramid multi‐scaling and channel attention mechanism to highlight salient features at each stage.
3. Considering that the lesion boundary is essential to precision segmentation, we propose a wavelet edge loss function, which uses wavelet decomposition to extract multi‐directional edge information of the lesion area to improve the accuracy of segmentation.

We apply the model to 4369 CT images obtained from 36 patients, and there are additional 2 cases in October which are co‐infected with underlying disease of tuberculosis. This type of coinfection symptom has also been reported in [20, 21] recently. Quantitative experiments demonstrate that our model achieves advanced performance in the segmentation of three symptoms and even to the coinfection condition.

## 2 RELATED WORK

In this section, we discuss three types of related work, including image semantic segmentation, medical image segmentation, and COVID‐19 image segmentation.

### 2.1 Image semantic segmentation

Image semantic segmentation is a very important research field in computer vision, which has a wide range of applications such as autonomous driving, medical image segmentation and three‐dimensional reconstruction. And many advanced image segmentation networks have been proposed so far. Fully Convolutional Network (FCNs) [22] modified the classification neural network, and introduced an end‐to‐end fully convolutional mechanism for semantic segmentation to achieve pixel‐level prediction. U‐Net [23] was a classic encoder and decoder structure, which was widely used in the medical field. Many subsequent networks were improved based on U‐Net, such as Attention U‐Net [24], which added an attention mechanism on the basis of U‐Net to focus the attention on important areas. UNet++ [25] connected the four layers of U‐Net together, allowing the network to learn the importance of features of different depths by itself. And [26] extended DenseNet to a fully convolutional network to achieve semantic segmentation. V‐Net [27] provided a three‐dimensional image segmentation method, and introduced a new objective function to deal with the problem of extreme imbalance between foreground and background. DeepLabv1 [28] combined a deep convolutional neural network and a probabilistic graph model, and used a dilated convolution algorithm to expand the receptive field to obtain more contextual information. Moreover, it used the fully connected conditional random field (CRF) [29] to improve the model's ability to capture details. DeepLabv2 [30] added an Atrous Spatial Pyramid Pooling (ASPP) model on the basis of v1. ASPP used atrous convolutions with different rates to capture multi‐scale contextual information of the image. DeepLabv3 [31] modified the ASPP module. DeepLabv3+ [32] introduced a classic encoder‐decoder structure on the basis of DeepLabv3. Pyramid Scene Parsing Network (PSPNet) [33] proposed a hierarchical global prior structure named pyramid pooling module, which contained information among different scales and sub‐regions, it combined four different pyramid scale features.

### 2.2 Medical image segmentation

Medical image segmentation can extract accurate, repeatable and quantitative pathological and physiological data to meet the needs of different biomedical researches and clinical applications. Therefore, image segmentation is widely used in various fields of medicine to assist in the diagnosis of diseases. Xiang et al. [34] proposed an NLCE module to combat disturbances, this module aggregated global context information and spatial dependencies to improve the robustness of medical image segmentation models. Cheng et al. [35] proposed a domain‐adaptive model called SIFA, which solved the domain shift problem from two aspects of feature and image under unsupervised conditions. Zhao et al. [36] proposed an automatic data augmentation model that could use the labelled data to synthesize other annotations, thereby saving a lot of time. Qin et al. [37] proposed a self‐focusing convolution structure to generate more detailed features through the dilated convolution and attention mechanism. The impact of diabetes on vision can be checked through the detection of digital retinal images, thereby reducing the risk of blindness in patients. Jen et al. [38] proposed a ten‐layer convolutional neural network to automatically discriminate and segment haemorrhages, exudates, and micro‐aneurysms. Gaál et al. [15] proposed a new lung segmentation method that used an adversarial critic model and an advanced fully convolutional network, this model had good robustness on different datasets. Ref. [16] used two deep convolutional neural networks for lung segmentation and solved the problem of dense abnormalities in chest X‐rays.

### 2.3 COVID‐19 image segmentation

Recently, there have been many studies on COVID‐19 infection segmentation. Fan et al. [39] proposed a new weakly supervised learning method to conduct a binary segmentation experiment on the public dataset COVID‐SemiSeg. And the network combined FCNs to perform multi‐class segmentation. Wang et al. [40] proposed a new model called COPLE‐Net, it exploited a self‐ensembling framework and a noise‐robust Dice loss to improve the segmentation capability. And 76,250 CT slices from 558 patients were used for experiments, the dice coefficient is 0.8072. Another deep learning model, named MiniSeg, proposed by Yu et al. [41], only had 472K parameters and could be quickly retrained by other COVID‐19 datasets. The DSC in this paper achieved 0.7728. Wu et al. [42] proposed the JCS system, which included a classification model and a segmentation model. The classification model was used to judge whether the people is sick, and the segmentation model was used to determine the location of the lesion. The dataset was 3855 CT slices from 200 patients and the dice similarity coefficient was 0.783. The authors of [43] proposed a model called EDANet, which used a dual attention module of channel and position with low computational complexity. This paper also constructed the first web‐based application to detect COVID‐19 from CXR images, which could help clinicians make diagnosis through any device. Yan et al. [44] introduced a feature variation (FV) block in the proposed network to enhance feature representation capability and segmentation performance. The DSC reached to 0.726 of 21,658 CT slices from 861 patients. Ref. [45] used the deep convolutional neural network and medical knowledge to diagnose early COVID‐19 in chest X‐rays. It employed 316 X‐ray images, of which 253 were positive. The proposed method achieved superior performance of 96% accuracy. Laradji et al. [46] proposed a weakly supervised learning algorithm, which performed point‐level annotations on CT images and used a consistency‐based loss function for training. The dice coefficient obtained is 0.73.

The proposal of classic segmentation networks and their applications in the medical field provide theoretical support for the research of COVID‐19. As shown above, many COVID‐19 segmentation networks have been proposed. However, most studies have not performed multi‐class segmentation. In this paper, we detect three types of disease: ground glass opacities, interstitial infiltrates and consolidation. And we propose a new deep learning model which employs pyramid attention and a wavelet edge loss function to improve the accuracy of lesion segmentation. Experiments have shown that our method achieves advanced performances.

## 3 PROPOSED METHOD

In this section, we first introduce the overall network architecture, and then show the core components in detail, including the pyramid attention module and the residual convolution module. Finally, we introduce our loss function.

### 3.1 Network architecture

**FIGURE 2:**
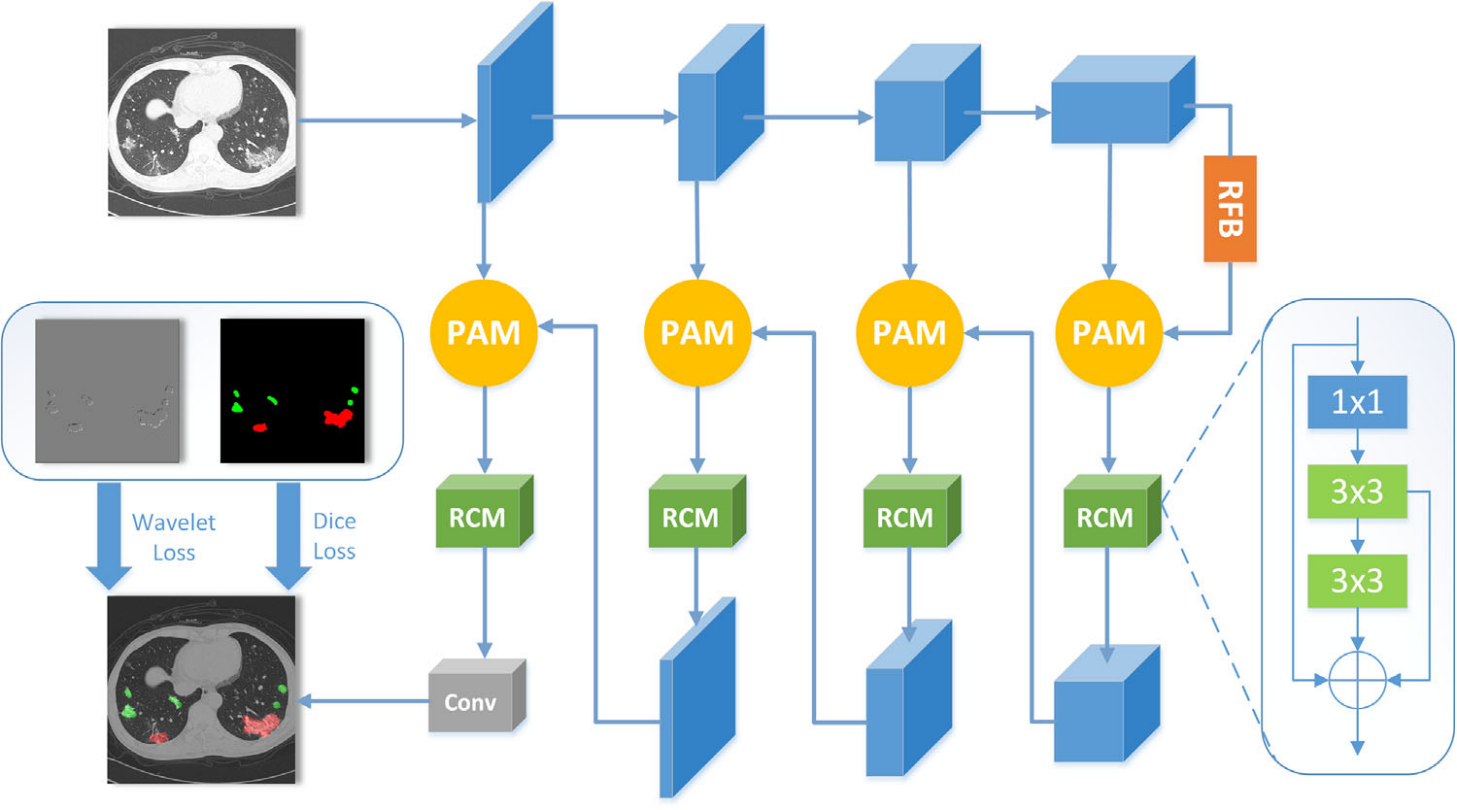
The architecture of the proposed model

The architecture of our network is shown in Figure 2. We use the first four stages of resnet101 [18] as the backbone. At each stage, first, we combine the feature map obtained from backbone with the one from the deeper scale stage. Then the concatenated feature is fed to a pyramid attention module (PAM) which combines the atrous spatial pyramid pooling module [31] and the channel attention module [48]. PAM can focus on salient areas and ignore irrelevant background feature responses by extracting multi‐scale context information. Second, the feature obtained from PAM is fed to a residual convolution module (RCM) which can improve the segmentation accuracy of the network. And the output of RCM of each stage is up‐sampled and forwarded to PAM of the previous stage. We pass the feature of the fourth stage to the receptive field module [47] (RFB), which can extract multi‐scale semantic information and expand the receptive field of feature maps. The detail parameters of the proposed network are shown in Table 1, and the digital represents the corresponding stage.

**TABLE 1:** The detail parameters of the proposed model

**Stage**	**Component**	**Output shape**
4	Res‐block4	32,32,1024
	PAM4	32,32,2048
	RCM4	32,32,512
	RFB	32,32,1024
3	Res‐block3	64,64,512
	PAM3	64,64,1024
	RCM3	64,64,256
2	Res‐block2	128,128,256
	PAM2	128,128,512
	RCM2	128,128,64
1	Res‐block1	256,256,64
	PAM1	256,256,128
	RCM1	256,256,32
	Conv	512,512,4

### 3.2 Pyramid attention module

**FIGURE 3:**
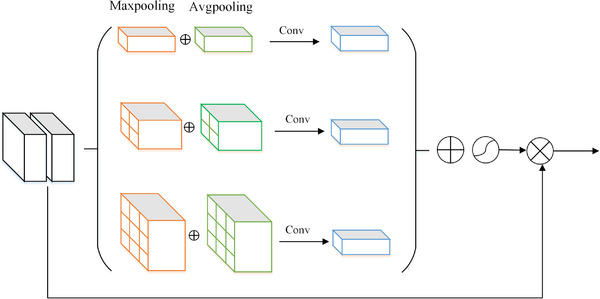
The architecture of the pyramid attention module (PAM)

The architecture of the pyramid attention module is shown in Figure 3. The two inputs of this module are the feature map of the current stage and the deeper stage. First, the concatenated feature map is respectively fed to the maximum and average pooling layer of 1 × 1, 2 × 2, 3 × 3, then we add the output feature map of maximum pooling and average pooling. Second, the feature map of each scale is passed through the convolutional layer to generate a C × 1 × 1 channel‐weight feature map. Finally, the three weight feature maps are combined and normalized by sigmoid, then the output is multiplied with the original feature map to focus the network's attention on important channels.

### 3.3 Residual convolution module

The residual convolution module is illustrated in Figure 2. This module is improved from the residual block of resnet101 [18]. The feature map goes through the 1 × 1, 3 × 3, 3 × 3 convolutions. Then the original feature and the output features of 3 × 3 layers are added together. The improvement we propose can extract more semantic information and enhance the ability to express features.

### 3.4 Loss function

We propose a new loss function, including a dice loss and a wavelet loss. The dice loss can compare the degree of overlap between the experimental result and ground truth. And we construct a wavelet loss function. Wavelet transformation [19] has multi‐resolution decomposition capability, which can extract multi‐level image features. And it has excellent expression ability for high‐frequency information, especially for edge details. We perform two‐dimensional discrete wavelet transformation on the predicted lesion area to obtain three components. They are the horizontal low‐frequency and vertical high‐frequency component LH, the horizontal high‐frequency and vertical low‐frequency component HL, the horizontal high‐frequency and vertical high‐frequency component HH. And we perform L1 loss for each component. The wavelet loss function can extract the edge information of the lesion, and deal with the segmentation difficulty caused by blurred and irregular boundaries. Our loss function is defined as follows,

(1) $$L\;\left(X_{i},Y_{i}\right)=\sum_{i=1}^{N}L_{dice}\left(X_{i},Y_{i}\right)+\lambda L_{wave}\left(X_{i},Y_{i}\right)\;$$

(2) $$L_{dice}\;\left(X_{i},Y_{i}\right)=\;1-\frac{2\left|X_{i}\cap Y_{i}\right|}{\left|X_{i}\right|+\left|Y_{i}\right|}$$

(3) $$\begin{array}{ccc} L_{wave}\;\left(X_{i},Y_{i}\right) & = & L_{1}\;\left(X_{i}^{LH},Y_{i}^{LH}\right)+L_{1}\left(X_{i}^{HL},Y_{i}^{HL}\right)\\ & & +\;L_{1}\left(X_{i}^{HH},Y_{i}^{HH}\right) \end{array}$$

where $\lambda =0.5,L_{1}\left(X,Y\right)={∥X-Y∥}_{1}$ , *N* is the number of CT slices, *X* is the predicted result, and *Y* is the ground truth.

## 4 EXPERIMENTS

In this section, first, the dataset and implementation detail are introduced, then we conduct ablation experiments and compare our model with other advanced methods. The results are shown in the figure and table below.

### 4.1 Dataset

In this paper, we use two datasets for experiments, one is the public dataset COVID‐SemiSeg [49], which includes 100 CT slices from 60 patients and three kinds of labels: ground‐glass, consolidation and pleural effusion. In the experiment, 50 slices are used for training, 5 for validation, and 45 for testing.

The other dataset we constructed comes from The Affiliated Hospital of Qingdao University. There is a total of 4369 CT slices from 36 patients, of which 18 are infected and 18 are normal. And the male to female ratio is 15:21. The patients are between 6 and 66 years old, and 11 of them have mild symptoms, 4 have moderate symptoms, and 3 are severe. 3496 slices are used for training and the rest for testing. And we apply our model to another 2 cases newly infected in October which are co‐infected with underlying disease of tuberculosis and analyse the results in Section 5. Our labels are marked by professional doctors and divided into three types of disease: ground glass opacities, interstitial infiltrates and lung consolidation. The composition of our dataset is shown in Table 2. Moreover, we perform a series of data enhancement operations such as flipping, rotating, and scaling on the training data to increase the segmentation accuracy. Our dataset also has some limitations. The amount of data is not sufficient enough, and the distribution of the three types of lesions is unbalanced, and even cross‐overlap occurs, which increase the complexity of segmentation. In this case, the proposed method has excellent performance, reflecting the robustness of the network.

**TABLE 2:** The composition of the dataset

**Infection type**	**Number**
Ground glass opacities	1091
Interstitial infiltrates	1012
Consolidation	1120
Ground glass opacities and interstitial infiltrates	343
Ground glass opacities and consolidation	93
Interstitial infiltrates and consolidation	32
Three types	17
Normal	661
Sum	4369

### 4.2 Implementation and evaluation

Our proposed network is implemented on PyTorch framework. We use SGD optimizer to train the model with momentum of 0.9, and weight decay of 0.0005. The initial learning rate is set as 0.001, and it decays by 0.1 for every 30 epochs. We resize all the input CT images as 512 × 512. Our experiment is performed on the TITAN RTX GPU with batch size 8 and 5‐fold cross validation.

In this paper, we use three evaluation metrics to quantify the model's performance, they are Dice Similarity Coefficient (DSC), Sensitivity (Sen.) and Specificity (Spec.) respectively. DSC indicates the degree of overlap between the predicted area *X* and the ground truth *Y*, which can be calculated as

(4) $$DSC\;\left(X,Y\right)=\frac{2\left|X\cap Y\right|}{\left|X\right|+\left|Y\right|}\;$$

The DSC value is between 0 and 1. The larger the value, the higher the accuracy. Sensitivity refers to the probability of not being missed when diagnosing a disease, and Specificity refers to the probability of not being misdiagnosed when diagnosing a disease. They are calculated as

(5) $$\mathrm{Sensitivity}\;=\frac{TP}{TP+FN}\;$$

(6) $$\mathrm{Specificity}\;=\frac{TN}{TN+FP}\;$$

where *TP*, *TN*, *FP*, *FN* indicate true positive, true negative, false positive, and false negative respectively.

### 4.3 Ablation experiments

In this section, we evaluate the contribution of core components of our model. The ablation results can be seen in Table 3. We start from the backbone without using proposed modules, as shown in A (backbone). In B (backbone + RCM), we evaluate the performance of the residual convolution module by comparing with A. Then we extend PAM and RCM to the network together as shown in C (backbone + RCM + PAM). Finally, on the basis of C, we add the receptive field block to the last stage of the network to construct our final segmentation network.

1. Effectiveness of RCM: We investigate the contribution of the RCM. For the three disease types, we observe that B (Backbone + RCM) is improved by an average of 2% in DSC compared to A (Backbone). This performance demonstrates that the RCM can improve the ability of our model to accurately segment the lesion area.
2. Effectiveness of PAM: we also evaluate the contribution of the pyramid attention module. The DSC of three lesions in B is (0.7255, 0.7401, 0.8130) and C (Backbone + RCM + PAM) is (0.7642, 0.7733, 0.8251). And the sensitivity of B and C is (0.7344, 0.7520, 0.8358), (0.7604, 0.8031, 0.8304) respectively. Obviously, PAM has greatly improved the dice metric and sensitivity of the network, especially for GGO and interstitial infiltrates that are difficult to distinguish. Therefore, PAM is essential to precise segmentation.
3. Effectiveness of RCM+PAM: We explore the importance of the combination of RCM and PAM. In Table 3, the DSC of C is improved by (6.01%, 5.18%, 2.19%) on the basis of A for three type lesions. It can be clearly observed that RCM and PAM are two important components of our network to improve the performance.
4. Effectiveness of the wavelet edge loss: Finally, we investigate the contribution of the wavelet edge loss. The results D (Backbone + RCM + PAM + RFB) and E (w/o wavelet loss) in Table 3 show that the wavelet loss function can increase the DSC and Sen. by 2% on average. It can improve the segmentation accuracy of our model by exploiting the edge information.

**TABLE 3:** Results of ablation experiments

**Method**	**Infection type**	**DSC**	**Sen**.	**Spec**.
A (Backbone)	Ground glass opacities	0.7041	0.7300	0.9983
	Interstitial infiltrates	0.7215	0.7590	0.9955
	Consolidation	0.8032	0.8357	0.9983
B (Backbone + RCM)	Ground glass opacities	0.7255	0.7344	0.9988
	Interstitial Iinfiltrates	0.7401	0.7520	0.9968
	Consolidation	0.8130	0.8358	0.9985
C (Backbone + RCM + PAM)	Ground glass Oopacities	0.7642	0.7604	0.9988
	Interstitial infiltrates	0.7733	0.8031	0.9959
	Consolidation	**0.8251**	0.8304	**0.9989**
D Our Nnet (Backbone + RCM + PAM + RFB)	Ground glass opacities	**0.7704**	**0.7883**	**0.9989**
	Interstitial infiltrates	**0.7900**	**0.8223**	0.9968
	Consolidation	0.8241	**0.8369**	0.9987
E Our net (w/o wavelet loss)	Ground glass opacities	0.7546	0.7801	0.9988
	Interstitial infiltrates	0.7750	0.7903	**0.9969**
	Consolidation	0.8043	0.7972	0.9989

### 4.4 Comparison with state‐of‐the‐art methods

To evaluate the infection segmentation performance, we compare our model with three current advanced networks: UNet++, Attention UNet and UNet‐CBAM which adds the classic attention module CBAM to UNet [48]. Quantitative results are shown in Table 4. As can be seen, the dice metrics output by UNet++ of the three disease types are 0.6955, 0.6994, 0.7880, respectively. As well as, the results are (0.7347, 0.7570, 0.8018) for Attention UNet and (0.7469, 0.7440, 0.8121) for UNet‐CBAM. For our model, the dice coefficients of the three types obtained are 0.7704, 0.7900, 0.8241. Obviously, the proposed network outperforms UNet++, Attention UNet, UNet‐CBAM in terms of DSC by a large margin, especially for GGO and interstitial infiltrates, which are also difficult for doctors to distinguish. And the *p*‐value between our model and UNet‐CBAM is $4.26\;\times 10^{-5}$, which presents the significance of the difference. For the sensitivity, our model also has excellent performance, and all models have similar level in terms of Specificity. We attribute the improvements to our pyramid attention module which highlights the salient features, residual convolution module which provides deeper and more detailed semantic information, and wavelet loss which focuses on edge information.

**TABLE 4:** Segmentation results of our model and other state‐of‐the‐art methods

**Method**	**Infection type**	**DSC**	**Sen**.	**Spec**.
UNet++ [25]	Ground glass opacities	0.6955	0.6591	0.9991
	Interstitial infiltrates	0.6994	0.7110	0.9956
	Consolidation	0.7880	0.7696	**0.9988**
Attention UNet [24]	Ground glass opacities	0.7347	0.7026	**0.9993**
	Interstitial infiltrates	0.7570	0.7756	0.9957
	Consolidation	0.8018	0.8126	0.9987
UNet‐CBAM [48]	Ground glass opacities	0.7469	0.7656	0.9988
	Interstitial infiltrates	0.7440	0.7768	0.9956
	Consolidation	0.8121	0.8199	**0.9988**
Our model	Ground glass opacities	**0.7704**	**0.7883**	0.9989
	Interstitial infiltrates	**0.7900**	**0.8223**	**0.9968**
	Consolidation	**0.8241**	**0.8369**	0.9987

**FIGURE 4:**
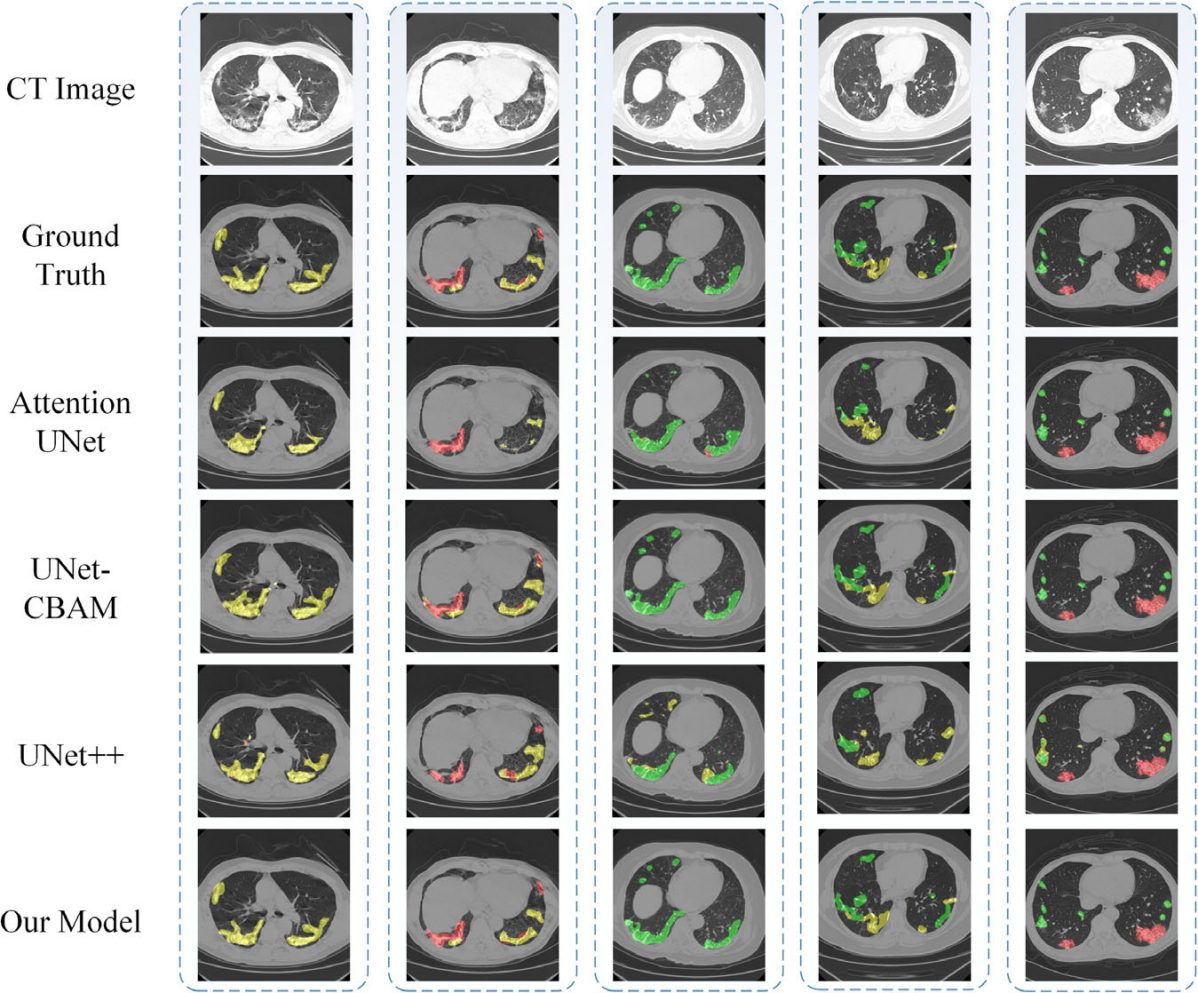
Some segmentation examples of our model and advanced methods, in which green, yellow, red represent three types of symptoms: ground glass opacities, interstitial infiltrates, and lung consolidation

And as shown in Figure 4, we provide some examples of different network segmentation results. The first row is the CT image and the second is the ground truth marked by professional doctors, and the following are result maps predicted by Attention UNet, UNet‐CBAM, UNet++ and our model respectively. It can be seen that our model can better deal with complex lesion areas.

### 4.5 Comparison with COVID‐19 segmentation methods

We also conduct the binary and multi‐class segmentation experiment on the public dataset COVID‐SemiSeg [49]. As shown in Table 5, for the binary segmentation, we only judge whether it is infected, and compare our model with some recently proposed methods: Semi‐Inf‐Net [17], MiniSeg [41] and CB‐PL [46] on the COVID‐SemiSeg dataset. The results present that our model achieves superior performance in terms of DSC, sensitivity, specificity. Moreover, we implement multi‐class segmentation based on the public dataset compared with DeepLab‐v3+ [32], FCN8s [22], Semi‐Inf‐Net and FCN8s [17], and Semi‐Inf‐Net and MC [17] for two types of lesions: ground glass opacities and consolidation, as shown in Table 6. The experiment results demonstrate that our model has better segmentation ability, and achieves significant improvement in three indicators especially for consolidation.

**TABLE 5:** Results of our model and recently proposed methods for binary segmentation on COVID‐SemiSeg dataset

**Model**	**DSC**	**Sen**.	**Spec**.
Semi‐Inf‐Net [17]	0.739	0.725	0.960
MiniSeg [41]	0.773	**0.836**	0.974
CB‐PL [46]	0.730	0.820	0.920
Ours	**0.779**	0.791	**0.983**

**TABLE 6:** Results of our model and other methods for multi‐class segmentation on COVID‐SemiSeg dataset

**Model**	**Ground glass opacities**			**Consolidation**		
	**DSC**	**Sen**.	**Spec**.	**DSC**	**Sen**.	**Spec**.
DeepLab‐v3+ [32]	0.443	0.713	0.823	0.238	0.310	0.708
FCN8s [22]	0.471	0.537	0.905	0.279	0.268	0.716
Semi‐Inf‐Net & FCN8s [17]	0.646	0.720	0.941	0.301	0.235	0.808
Semi‐Inf‐Net and MC [17]	0.624	0.618	0.966	0.458	0.509	0.967
Ours	**0.682**	**0.729**	**0.987**	**0.548**	**0.638**	**0.994**

## 5 DISCUSSION

In this study, we propose a multi‐class COVID‐19 segmentation network which designs a pyramid attention module that employs multi‐scale pyramid structure and channel attention mechanism with the fusion of maximum and average pooling to improve segmentation accuracy, and a residual convolution module that extracts richer semantic information. We also propose a wavelet loss to enhance the expression of edge features. Extensive experiments have presented that our model achieves higher performance than state‐of‐the‐art methods. The average DSC, sensitivity, and specificity of the three lesion types obtained from our network are 0.7948, 0.8158, 0.9981, respectively.

**FIGURE 5:**
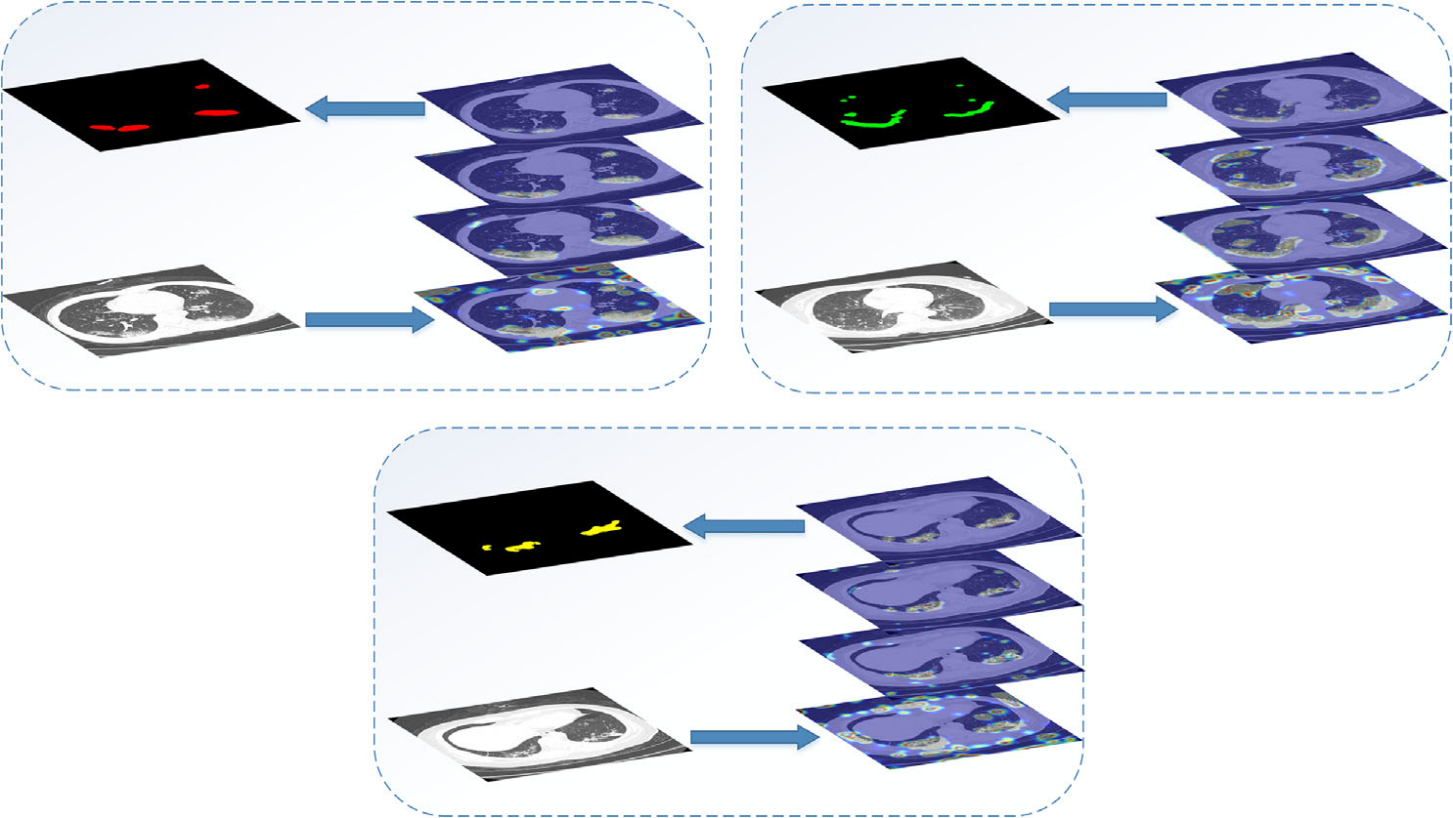
Visualization of our model, the four activation maps are obtained from four stages, and the bottom feature map is the output of the fourth stage

In order to understand the attention of the network more clearly and intuitively, we display the feature map output from each stage in the form of a heat map, as shown in Figure 5. We input three CT images with the lesion of ground glass opacities, interstitial infiltrates, and lung consolidation into the network. It can be seen from the figure that the attention of the network in the fourth stage is relatively scattered. And as the network decoder progresses, the network pays more attention to the lesion area, which reflects the effectiveness of our network segmentation capability.

**FIGURE 6:**
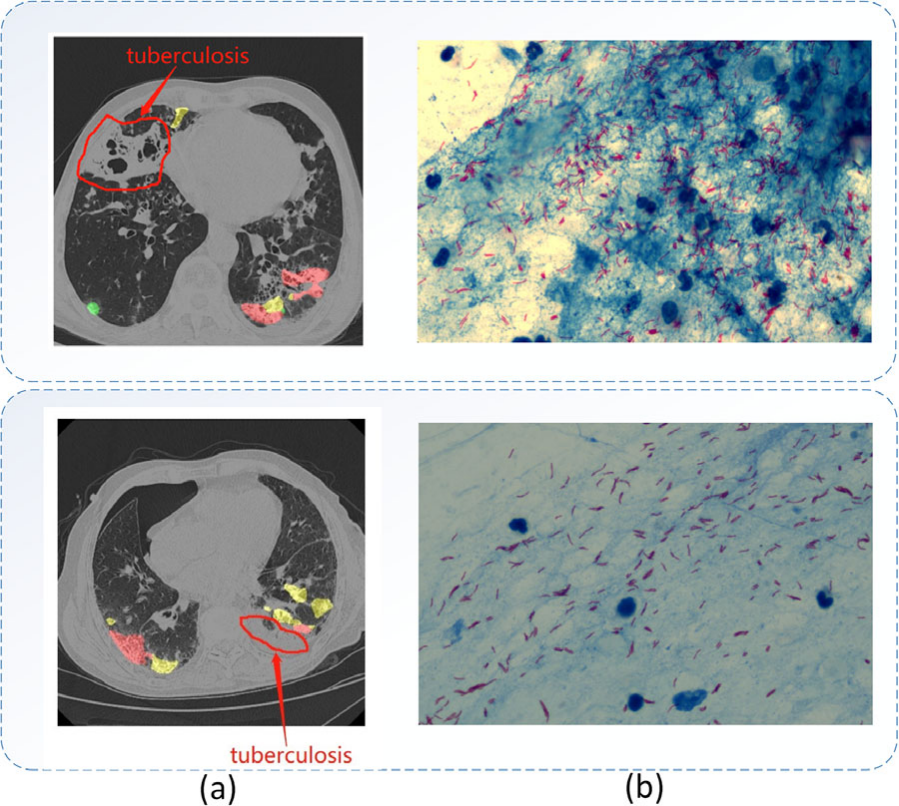
The COVID‐19 segmentation results with pulmonary tuberculosis. (a) The CT image with the tuberculosis lesion tag and segmentation results, (b) the detected mycobacterium tuberculosis (shown in red) by Ziehl–Neelsen stain

We apply our model on two COVID‐19 infection patients in October include coinfection with underlying disease of tuberculosis. The characteristics of CT images of pulmonary tuberculosis are that the lesions mostly occur in the apical posterior segment of the upper lobe, the dorsal and posterior segment of the lower lobe, showing polymorphism, infiltration, proliferation, caseous necrosis, fibrosis and calcification symptoms, which can be present at the same time. It is easy to form cavities and disseminated lesions, shown in the tags in Figure 6. The CT manifestations of COVID‐19 are characterized by viral pneumonia. In the early stage of the disease, there is ground glass opacity. During the progression of the disease, the lung lobe or pulmonary segment or even the whole lung may become consolidated. In the later stage of the disease, fibrous cords sometimes present.

We aim to study the COVID‐19 CT segmentation method and have not done any annotation on pulmonary tuberculosis lesions. The left column in Figure 6 shows the COVID‐19 segmentation results on the patients with pulmonary tuberculosis. And the right column is the detected mycobacterium tuberculosis (shown in red) by using the sputum smear for microscopic examination (acid‐fast bacilli is positive for acid‐fast staining, Ziehl–Neelsen stain) for the patients. The segmentation results also illustrate the robustness of our method in coinfection conditions.

## 6 CONCLUSION

In this paper, we propose a novel multi‐class COVID‐19 CT infection segmentation network, which designs a pyramid attention module and a residual convolution module to improve the identification of lesion areas. The pyramid attention module (PAM) combines the pyramid architecture on the attention mechanism to extract multi‐scale contextual information. And the residual convolution module can enhance the feature expression ability of the network. Moreover, we also provide a wavelet loss which exploits wavelet decomposition to alleviate the shortage of irregular, fuzzy infection region edges. And we diagnose three types of disease: ground glass opacities, interstitial infiltrates, and lung consolidation on 4369 CT slices. Extensive experiments have demonstrated that our network achieves the state‐of‐the‐art performance and is robust on the coinfection condition with underlying tuberculosis. Since the COVID‐19 segmentation dataset needs to be labelled by professional doctors, it is time‐consuming and labour‐intensive. Therefore, we plan to use weakly supervised learning to extend this work, and train the network with both labelled and unlabelled data. Such a method will be undoubtedly helpful for collecting segmentation data and conducting experiments.
